# Design, synthesis, and preliminary biological evaluation of catalpol propionates as antiaging drugs

**DOI:** 10.1186/s13065-019-0626-3

**Published:** 2019-08-21

**Authors:** Chunhong Dong, Shuanglin Liu, Xiaodong Cheng, Qiang Wang, Shiqing Jiang, Guoqing Wang

**Affiliations:** 1 Henan University of Chinese Medicine, Zhengzhou, 450046 Henan China; 2 Department of Applied Chemistry, Zhengzhou University of Light Industry, Zhengzhou, 450002 Henan China; 3 High & New Technology Research Center of Henan Academy of Sciences, Zhengzhou, 450002 Henan China

**Keywords:** Molecular docking, Synthesis, Catalpol propionate, Antiaging drug

## Abstract

In this paper, catalpol propionylated analogs (CPs) were designed as drug ligands of glutathione peroxidase (GSH-Px) based on molecular docking (MD) using Surflex-Docking method. The calculated total scores (Total_score) and C log *P* of CPs are higher than that of catalpol, which show that the CPs maybe served as potential lead compounds as new antiaging drugs. Furthermore, the maximum Total_score of isomers in one group CPs is often not that the molecule with minimum energy structure. These show that the CPs docking with GSH-Px maybe not only affected by the molecular energy, but also affected by their conformations. The CPs were synthesized by esterification of catalpol with propionic anhydride using pyridine as solvent and acid banding agent, DMAP as catalyst, reaction at specific temperature. The synthesized perpropionylated catalpol analog (CP-6) was determined by NMR, FT-IR, HRMS, and HPLC, and the synthesis process was optimized by means of orthogonal experimental design. Subsequently, CP-6 was screened for cells viability by MTT assay, the results show that the CP-6 can effectively reversed STZ-induced reduction of cells viability, and CP-6 has potential antiaging activity.

## Introduction

Aging is closely associated with diverse chronic diseases, such as hypertension, diabetes, and multiple cancers, etc. [1–3]. With the trend of global aging and the improvement of living standards, health problems caused by aging and aging-associated diseases have become increasingly prominent [2, 3]. It is well accepted that the most significant determinant of aging is oxidative damage caused by overproduction of reactive oxygen species (ROS) [4–6]. Meanwhile, ROS is expected to play an additional role in aging by directly or indirectly damaging the glutathione peroxidase (GSH-Px) and other antioxidase [7]. More and more evidences demonstrate that there is a positive relationship between aging and immune dysfunction [8–10]. The agents with anti-oxidant and/or immunomodulatory functions might be of preventive or potential therapeutic value in aging-associated diseases [11].

The therapeutic efficacy of biologically active natural compounds offers useful platform for developing new antiaging drugs [12, 13]. Catalpol is belong to iridoid glucoside, richly in roots of *Rehmannia glutinosa Libosch* [14]. It is well known that catalpol has multiple pharmacological activities, including anti-inflammatory and anti-oxidative effects [14–24]. However, there is no reported that catalpol used as clinical drug in the treatment of age-related diseases because of its blood–brain barrier caused by low lipophilicity and fast metabolism in body [9]. It is necessary to improve its lipophilicity, reduce its blood–brain barrier, increase its utilization in vivo, and modify its structure for used as antiaging drugs.

Molecular docking (MD) is involved in placing the target compound (ligand) into the binding site of a receptor, and finding the appropriate binding position within the receptor. Surflex-Dock was applied to perform MD using empirical scoring functions and a patented search engine to dock ligand into a protein’s binding site [25, 26]. Surflex-Dock scores (Total_score) were expressed in −log10 (K_d_) units to represent binding affinities, and the compounds with higher Total_score can be used as potential antiaging drugs.

In this paper, Surflex-Dock was used to investigate the docking binding affinities of the designed catalpol propionylated analogs (CPs) with GSH-Px. Total_scores of the designed CPs with different molecular energy and conformations were calculated, and the binding modes between CPs and GSH-Px were visualized. The CPs were synthesized by esterification of catalpol with propionic anhydride in pyridine as solvent and acid banding agent, and the synthesized perpropionylated catalpol analog (CP-6) was preliminarily screened for antiaging activity by MTT assay.

## Materials and methods

Catalpol was extracted from Rehmannia glutinosa in laboratory, purity is 98%. All the chemicals and organic solvents used in the synthetic process are AR grade. Propionic anhydride, pyridine, sodium bicarbonate and anhydrous sodium sulfate were purchased from Shanghai Alpha Chemical Co., Ltd., China. Methylsilicone oil was purchased from Changzhou Longcheng Organosilicon Co., Ltd., China. Chromatographic grade methanol and acetonitrile were purchased from Tianjin Siyou Fine Chemicals Co., Ltd., China. HPLC were performed using Waters-E2695 (Waters Inc., USA). High resolution mass spectra (HRMS) were recorded by Thermo Fisher-Exactive Obitrap Mass Spectrometer (Thermo Fisher Inc., USA). NMR spectra were recorded on Agilent 400 MHz NMR spectrometer (Agilent Inc., USA). FT-IR spectra were measured on Nicolet iS5 (Thermo Fisher Inc., USA). Solvent CDCl_3_ used in NMR were obtained from Aldrich Chemical Co., Inc. RE-2000B rotary evaporator is purchased from Zhengzhou Kaipeng Experimental Instrument Co., Ltd., China. Ultrapure water was prepared using Milli-Q Century (Millipore Company, USA).

### Structure based antiaging drug design

It has been reported that GSH-Px is one of the most important target enzyme for most antiaging drugs. Therefore, GSH-Px was selected to conduct molecular docking with the designed CPs and predict their antiaging activity according to the consistency scoring function. Tripos force field was utilized for energy minimization, and followed by protomol generation [27]. The protomol was created by extracting the original ligand (PDB ID: 2F8A). All crystal water and small molecular ligands in protein crystals were removed. Hydrogen atoms with essential H-bond orientation and charge were added. In Multi-Channel Surface mode, several active pockets were generated and one of the best docking active pockets is selected, and the relevant side chains were repaired, then the binding mode of the CPs and GSH-Px was calculated by adopting an empirical scoring function and a patented searching engine. The threshold is set to 0.50, and the expansion coefficient is set to 1 to form a prototype molecule for docking in high-precision mode. The original ligand in the active pocket as a reference was used to carry out the docking operation between the receptor and the ligand sets. Subsequently, a series of isomers of CPs were docked at the virtual active site via Geom X method by considering every ligand [28]. The binding affinity of the ligands is predicted in terms of Total_score which is expressed as −log10Kd, where Kd is binding constant.

### Procedure for synthesis, characterization and purification of CPs

**Scheme 1:**
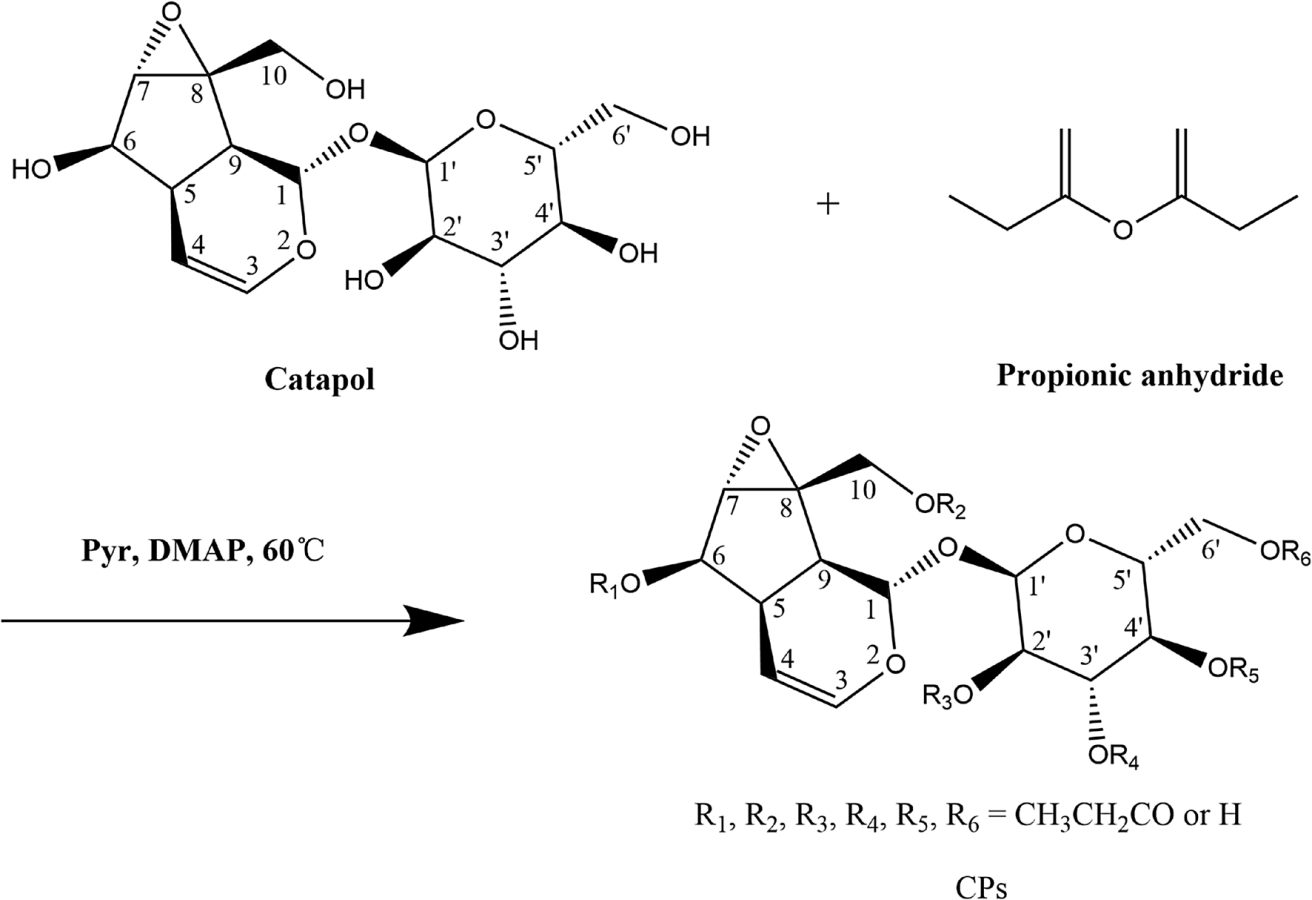
Synthetic route of CPs

The synthesis route of CPs is shown as Scheme 1 [29, 30], in which R_1_, R_2_, R_3_, R_4_, R_5_, R_6_=CH_3_CH_2_CO/–H, and the number of all the possible isomers is 71.

All the reagents and solvents used in the reaction were dried in the conventional way. To a stirred solution of catalpol (100 mg, 0.27 mmol) in pyridine (5 mL) was added propionic anhydride (0.6 mL, 4.7 mmol). The resulting mixture was stirred and reacted at 60 °C.

HPLC was used to monitor the reaction process and the reaction was terminated until the peak of catalpol was disappeared. The HPLC conditions were set as following: PDA detector (210 nm), Agilent-C18 chromatographic column (1250 × 4.6 mm), column temperature 25 °C, mobile phase as acetonitrile:water (V/V) = 70:30.

After the reaction ended, the mixture was poured into ice water and extracted with 60 mL CH_2_Cl_2_ three times. The combined organic layers were washed three times with 90 mL of saturated sodium bicarbonate solution, then dried over with MgSO4 and concentrated.

In order to obtain the optimal reaction conditions in the propionylation of catalpol, the molar ratio of reactant (A), reaction temperature (B), reaction time (C), and concentration of pyridine (D) were set as the variable parameter of synthetic reaction. Conversion rate of CP-6 was set as the target index, and four factors in three levels (shown as Table 1) experiments were carried out and visual analyzed according to the orthogonal experiment design $\mathop {\text{L}}\nolimits_{ 9} \left( {\mathop 3\nolimits^{ 4} } \right)$.

**Table 1:** Factor and level of the synthesis process

A (mol/mol)	B (°C)	C (h)	D (mg/L)
Factor			
6:1	30	12	15
9:1	60	24	20
12:1	90	48	25

### MTT assay for cells viability

SH-SY5Y human neuroblastoma cells are related to aging, and the viability of SH-SY5Y cells were detected by MTT (3-(4, 5-dimethylthiazol-2-yl)-2, 5diphenyl tetrazolium bromide) assay [31]. Cells were seeded overnight at a density of 10^5^ cells/well 96-well plates in 100 μL medium. The Cells were co-incubated with 20 μM CP-6 with streptozotocin (STZ, 0.8 μM) for 24 h. Then, the medium was removed and added 0.5 mg/μL MTT. After incubation at 37 °C for 4 h, 100 μL of dimethyl sulfoxide (DMSO) was added to SH-SY5Y cells each well, and the mixture was shaken at a low speed for 15 min to fully dissolve the formazan crystals, followed by the measurement of the absorbance at 490 nm using an SpectraMax i3x spectrophotometer (Molecular Devices, USA).

## Results and discussion

### Molecular docking study

**Scheme 2:**
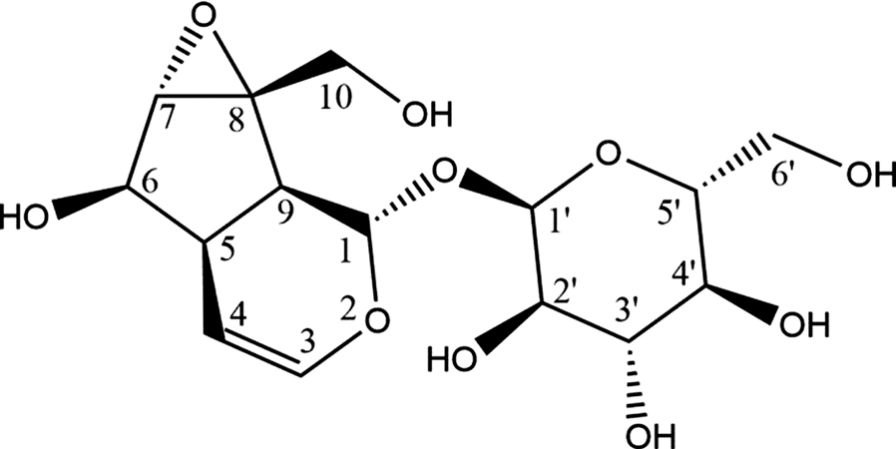
Structure of catalpol

**Fig. 1:**
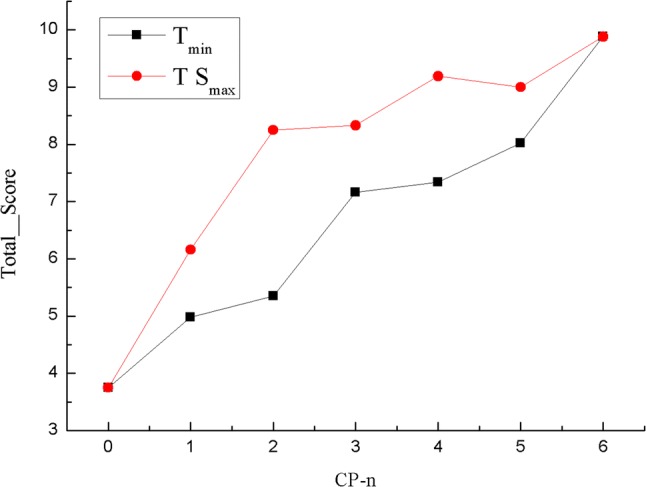
Total score of isomers of the CPs in different compound groups. n = 0, 1, 2, 3, 4, 5, and 6 respectively represents the number of propionylated hydroxyl groups

The structure of catalpol was shown as Scheme 2. It can be seen that there are six hydroxyl groups in its molecule structure, and their positions are indicated as 2′, 3′, 4′, 6′, 6, and 10, respectively. In order to improve its blood–brain barrier and fast metabolism, one to six of the hydroxyl groups were designed to be propionylated as CPs. The designed CPs were docked with GSH-Px using Surflex-Docking method, and the calculated Total_score are shown in Table 2. The Total_score of the isomers of the CPs in different compound groups with the minimum energy (Emin) and maximum value (TSmax) are shown as Fig. 1. The lipophilicity (C log *P*) of the CPs evaluated by in silico calculation based on their chemical structures were also shown in Table 2.

**Table 2:** Total score of CPs docked with GSH-Px and their C log *P*

Number^1^	Position^2^	Total_score	C log *P*	Position^2^	Total_score	C log *P*
0	–	3.75	− 4.38			
1	2′	4.04	− 3.36	6′	5.31	− 3.60
	3′	6.16	− 3.00	10^3^	4.98	− 2.95
	4′	5.00	− 4.31	6	4.70	− 2.95
2	4′, 6′	5.28	− 2.88	4′, 10	5.67	− 2.88
	2′, 6′	4.83	− 2.88	2′, 3′	5.68	− 2.28
	3′, 6′	5.24	− 2.23	3′, 6	6.14	− 1.57
	6′, 10^3^	5.35	− 2.17	3′, 10	6.11	− 1.57
	6′, 6	5.93	− 2.17	2′, 10	8.25	− 2.22
	3′, 4′	5.72	− 2.93	2′, 6	5.56	− 2.22
	2′, 4′	7.80	− 2.93	6, 10	5.24	− 1.52
	4′, 6	5.24	− 2.88			
3	4′, 6′, 10	6.73	− 1.45	2′, 4′, 6	6.51	− 1.50
	3′, 4′, 6′	6.55	− 1.50	2′, 3′, 4′	6.73	− 1.55
	2′, 4′, 6′	6.08	− 1.50	3′, 4′, 6	6.38	− 1.50
	4′, 6′, 6	7.69	− 1.45	3′, 4′, 10	6.19	− 1.50
	3′, 6′, 6	8.33	− 0.80	4′, 6, 10	7.15	− 1.45
	3′, 6′, 10	7.65	− 0.80	3′, 6, 10	5.74	− 0.14
	2′, 3′, 6′	6.41	− 1.50	2′, 3′, 6	6.24	− 0.85
	2′, 6′, 6	6.96	− 1.45	2′, 3′, 10	6.48	− 0.85
	2′, 6′, 10	6.67	− 1.45	2′, 6, 10	6.58	− 0.80
	2′, 4′, 10	6.25	− 1.50	6′, 6, 10^3^	7.16	− 0.74
4	2′, 6′, 6, 10	7.25	− 0.02	2′, 4′, 6, 10	6.56	− 0.07
	2′, 3′, 4′, 6′	5.31	− 0.12	3′, 4′, 6, 10	8.06	− 0.07
	3′, 4′, 6′, 6	7.45	− 0.07	2′, 3′, 6, 10	7.18	0.58
	3′, 4′, 6′, 10	9.19	− 0.07	2′, 4′, 6′, 6	6.62	− 0.07
	2′, 3′, 6′, 10	7.15	− 0.07	2′, 4′, 6′, 10	8.15	− 0.07
	2′, 3′, 6′, 6	5.92	− 0.07	4′, 6′, 6, 10^3^	7.34	− 0.01
	2′, 3′, 4′, 6	5.46	− 0.12	3′, 6′, 6, 10	6.35	0.64
	2′, 3′, 4′, 10	7.36	− 0.12			
5	2′, 3′, 6′, 6, 10	7.90	1.36	2′, 4′, 6′, 6, 10	8.02	1.36
	2′, 3′, 4′, 6′, 6	7.38	1.31	2′, 3′, 4′, 6, 10	7.89	1.31
	2′, 3′, 4′, 6′, 10	9.00	1.31	3′, 4′, 6′, 6, 10^3^	7.97	1.36
6	2′, 3′, 4′, 6′, 6, 10	9.88	2.74			

^1^Number of propionylated hydroxyl groups in CP

^2^Positions of propionylated hydroxyl groups in CP

^3^The isomer with minimum molecular energy

It can be seen from Table 2 that the Total score are obviously increased with the increase of the number of propionylated hydroxyl groups. The Total_scores of the CPs are all higher than that of catalpol, and the Total_score reaches maximum when the number of propionylated hydroxyl group is six. It also can be seen from Table 2 that the C log *P* are also increased with the increase of the number of propionylated hydroxyl groups, which indicates that the lipophilicity increased while propionylation of the hydroxyl groups in catalpol. These results indicate that propionylation can improve the antiaging activity of catalpol analogs, and maybe that CP-6 is the best one.

Meanwhile, it can be seen from Fig. 1 that the maximum Total_score in one group isomers is not the CP with the minimum energy of molecule structure. This indicates that the pharmacological activity and chemical stability of CPs maybe different, and suitable synthesis conditions should be selected for producing pharmacological active structure.

**Fig. 2:**
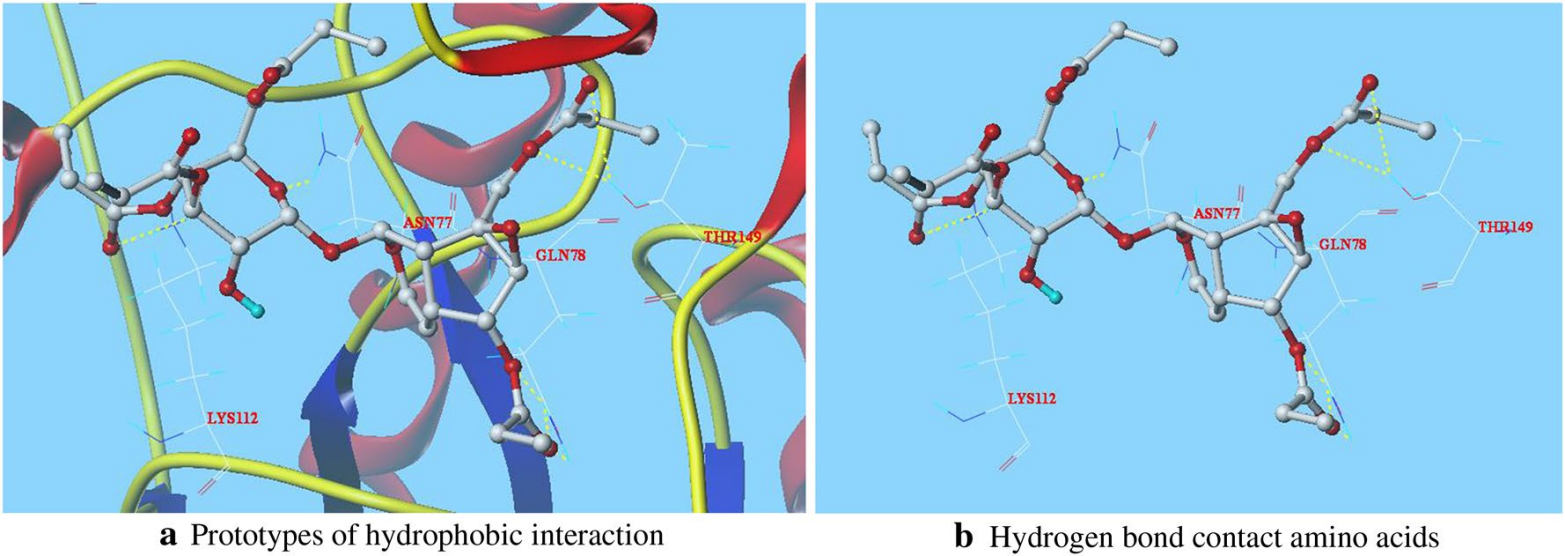
Docking conformation of CP-5 with GSH-Px at the active site

**Fig. 3:**
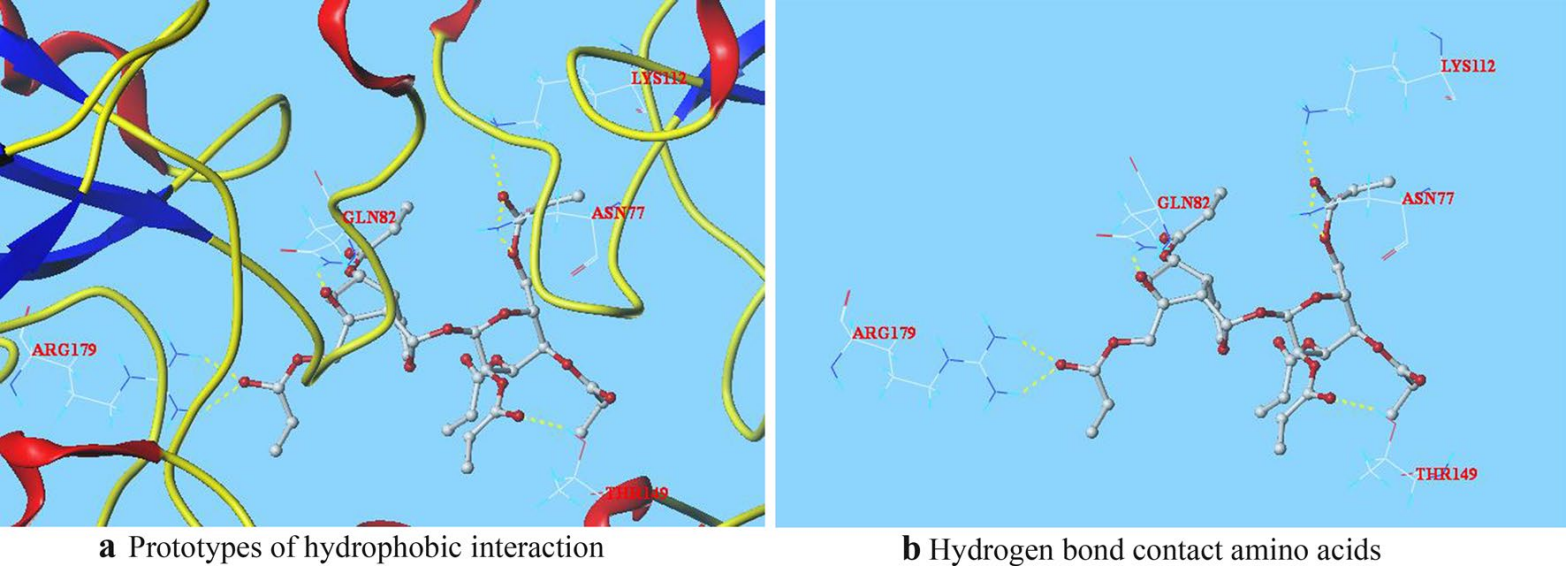
Docking conformation of CP-6 with GSH-Px at the active site

The patterns of interaction between CP-5, CP-6, which Total score are higher than seven and C log *P* are higher than 1.3, and GSH-Px are shown as Figs. 2 and 3, respectively. The rod-like molecules are amino acid residues, and the coarse globular small molecules are CPs. The red represents O, the white represents C, the blue represents N, and the indigo blue represents H. The fluorescent yellow dotted line is a hydrogen bond formed between the small molecules and the amino acid residues surrounding GSH-Px.

In Fig. 2, the oxygen at 4′-position in the carbonyl of CP-5 form two hydrogen bonds with residue LYS112. The 2-position oxygen on six-membered ring of CP-5 forms a hydrogen bond with hydrogen in the N–H bond of residue ASN77. Two oxygens at 6-position on five-membered ring in CP-5 form two hydrogen bonds with two hydrogens in the N–H bond of residue GLN78, and the oxygen in carbonyl at 6-position of CP-5 forms a hydrogen bond with another hydrogen in N–H bond of residue GLN78. Two oxygens at the 10-position of CP-5 form two hydrogen bonds with two hydrogens in the O–H bond of residue THR149.

In Fig. 3, the oxygen in the carbonyl at 2′-position on the sugar ring of CP-6 forms a hydrogen bond with hydrogen in the O–H bond of residue THR149. Two oxygens at 6′-position in the CP-6 forms two hydrogen bonds with hydrogens in the O–H bond of residue ASN77, respectively. The oxygen at 6′-position in the carbonyl of CP-6 forms a hydrogen bond with residue LYS112. The oxygen on three-membered ring of CP-6 forms a hydrogen bond with hydrogen in the N–H bond of residue GLN82. The oxygen in carbonyl at 10 position of CP-6 form two hydrogen bonds with two hydrogens in the N–H bond of residue ARG179.

### Synthesis of CPs and characterization of the synthetic process

#### Preparation of CPs

**Scheme 3:**
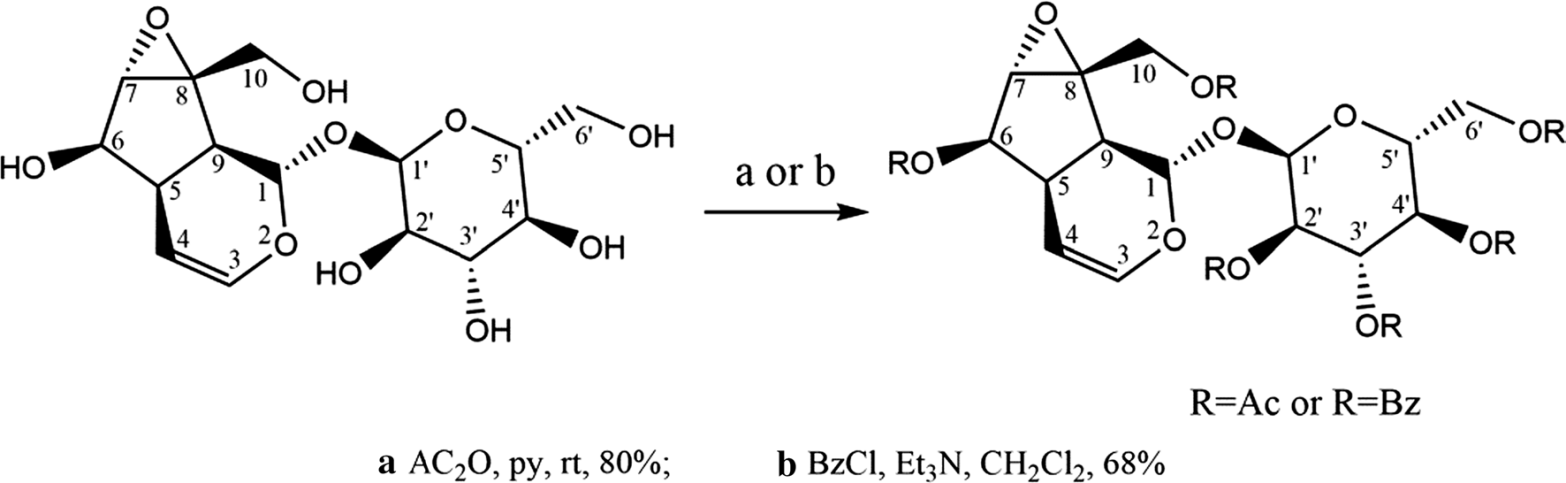
Standard synthesized method of peracetylated catalpol analogs

The first peracetylated catalpol analogs were synthesized by standard method, shown as Scheme 3 [32]. The designed CPs were tried to be synthesized by substitution of Ac_2_O with propioic anhydride.

However, no new product was found after the reaction monitored for 36 h. It indicates that the reaction was not carried out in the standard reaction conditions. In order to carry out synthesis process of CPs, DMAP was added as catalyst, and reaction temperature was adjusted to 60 °C. Subsequently, the designed CPs were obtained. In the synthesis process of CPs, pyridine is not only acts as a solvent, but also acts as an acid binding agent, and DMAP is used as catalyst. The improved standard method is shown as Scheme 1 [29, 30].

#### Determination of CP-6

**Fig. 4:**
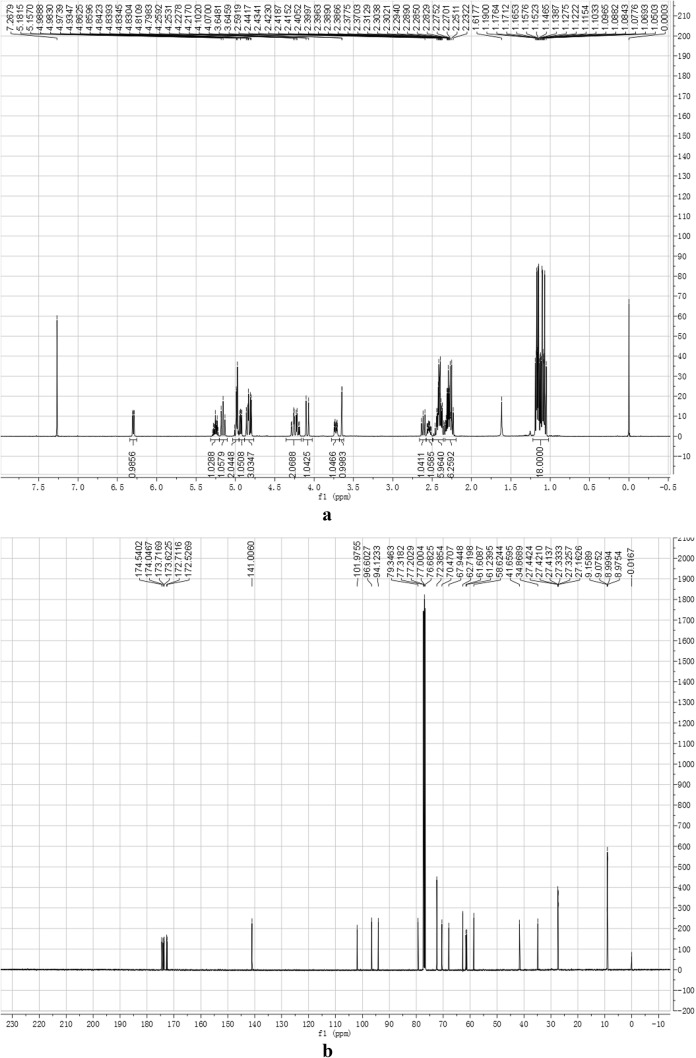
NMR spectra of CP-6. **a** ^1^H NMR spectrum; **b** ^13^C NMR spectrum

**Fig. 5:**
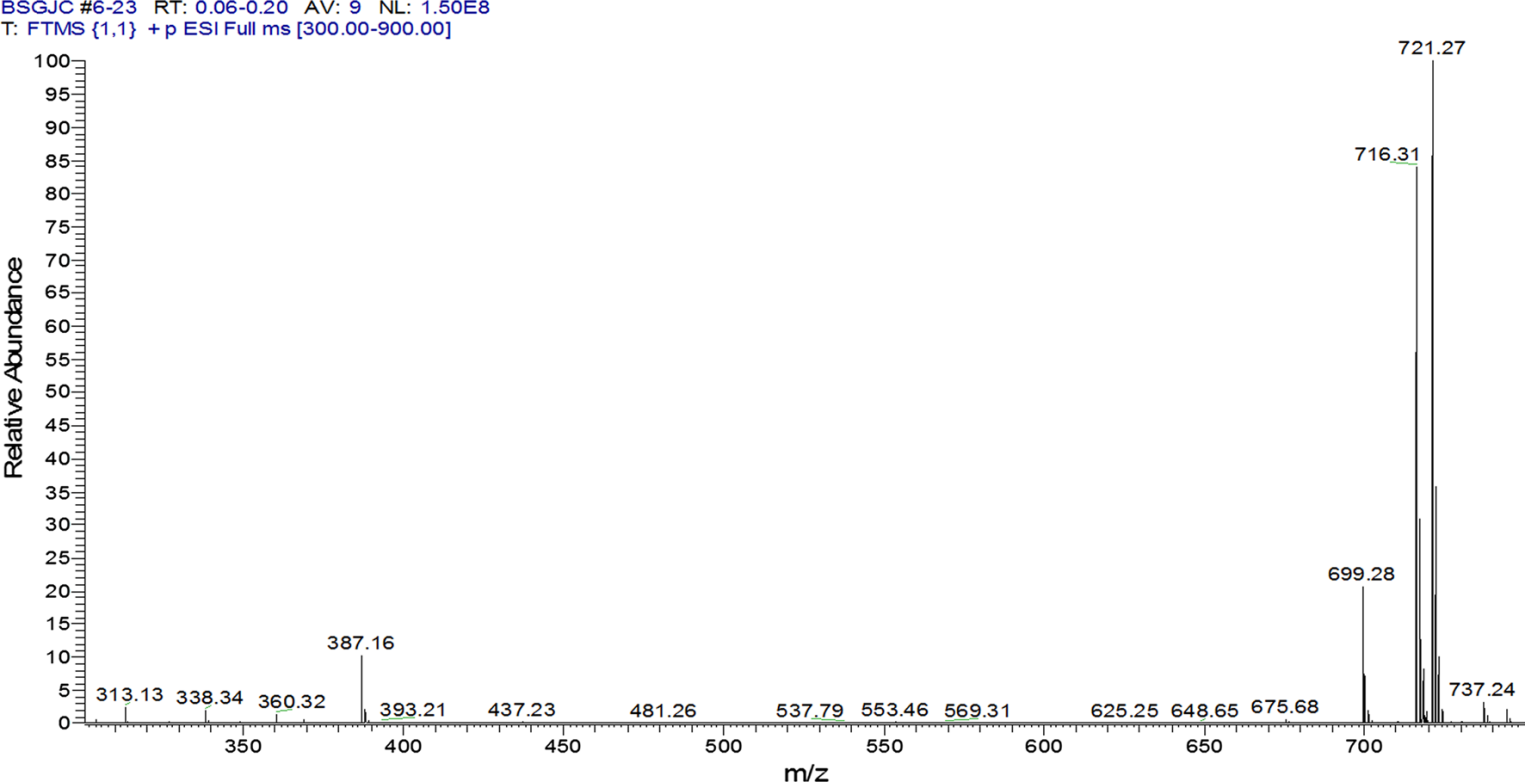
HRMS spectrum of CP-6

**Fig. 6:**
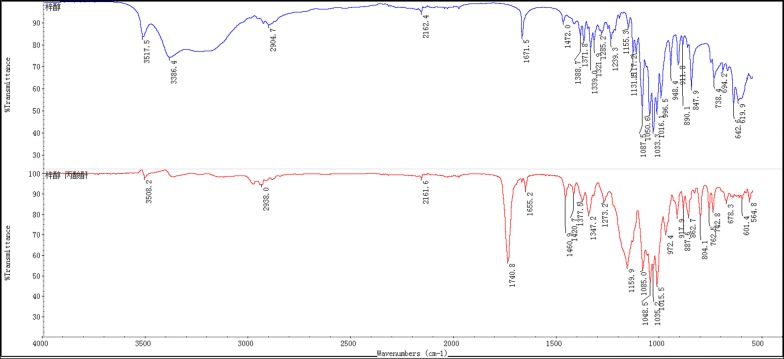
FT-IR spectra of catalpol (up half) and CP-6 (lower half)

The target product was prepared and confirmed by NMR, MS, IR. Its ^1^H NMR (400 MHz, CDCl_3_) and ^13^C NMR (100 MHz, CDCl_3_) were shown as Fig. 4a, b, respectively. In Fig. 4a, δ 6.30 (1H, dd, J = 1.7, 6.0 Hz, H-3), 5.25 (1H, m, H-3′), 5.16 (1H, t, J = 9.7 Hz, H-4′), 4.95–5.00 (2H, m, H-1′, 2′), 4.93 (1H, dd, J = 4.5, 6.0 Hz, H-4), 4.81–4.86 (2H, m, H-10a, 6), 4.80 (1H, d, J = 5.6 Hz, H-1), 4.10–4.26 (2H, m, H-6′), 4.07 (1H, d, J = 12.8 Hz, H-10b), 3.73 (1H, m, H-5′), 3.65 (1H, s, H-7), 2.61 (1H, dd, J = 7.8, 9.4 Hz, H-9), 2.54 (1H, m, H-5), 2.23–2.44 (12H, m, CH_2_ × 6), 1.02–1.21 (18H, m, CH_3_ × 6); In Fig. 4b, δ 174.54, 174.04, 173.71, 173.62, 172.71, 172.52, 141.00, 101.97, 96.60, 94.12, 79.34, 72.38, 70.47, 67.94, 62.71, 61.60, 61.23, 58.62, 41.65, 34.86, 27.44, 27.42, 27.41, 27.33, 27.32, 27.16, 9.15, 9.07, 8.99, 8.97. Its HRMS (ESI) m/z (pos) was shown as Fig. 5, where C_33_H_46_O_16_ M = 698.28, three main m/z peaks [M + H]^+^ 699.2864, [M + NH_4_]^+^ 716.3131, [M + Na]^+^ 721.2773. Compared the FT-IR spectra of catalpol (shown as Fig. 6, up half) and that of CP-6 (shown as Fig. 6, lower half), it can be seen there are absorptive peaks (cm^−1^) 3386.4 (OH), 1671.5 (C=C), 1050.6 (CH_2_OH) and 1131 (CHOH) in catalpol, while the absorptive peak of -OH at 3386.4 cm^−1^ was disappeared in CP-6, and a strong ester carbonyl absorptive peak was appeared at 1740.8. All above results indicate that the six hydroxyl groups in the catalpol are all esterified.

**Fig. 7:**
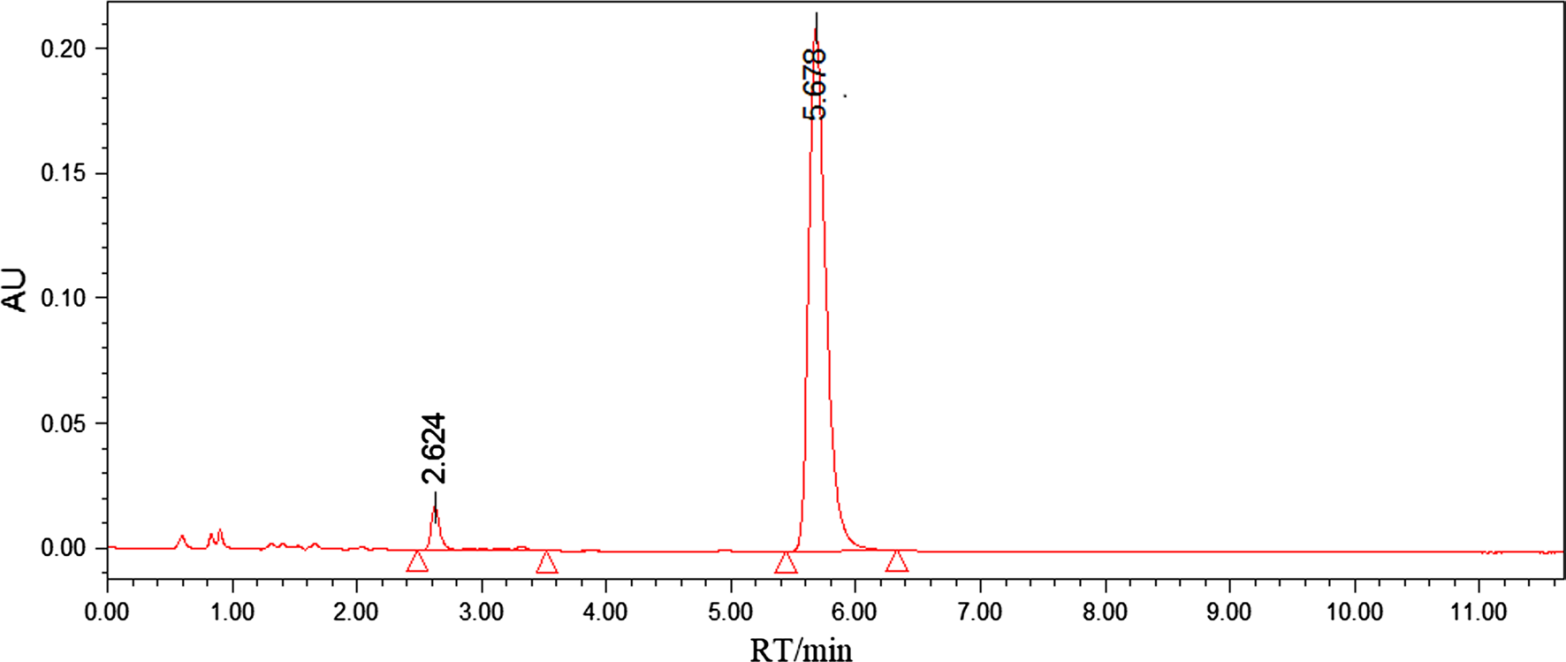
Determination of purified solid sample by HPLC

The residual of the purification process was crystallized from ethanol and obtained 121.27 mg yellow solid. The solid sample was determined by HPLC and shown as Fig. 7 with the purity calculated as 95.03%.

#### Characterization of the synthesis process

**Fig. 8:**
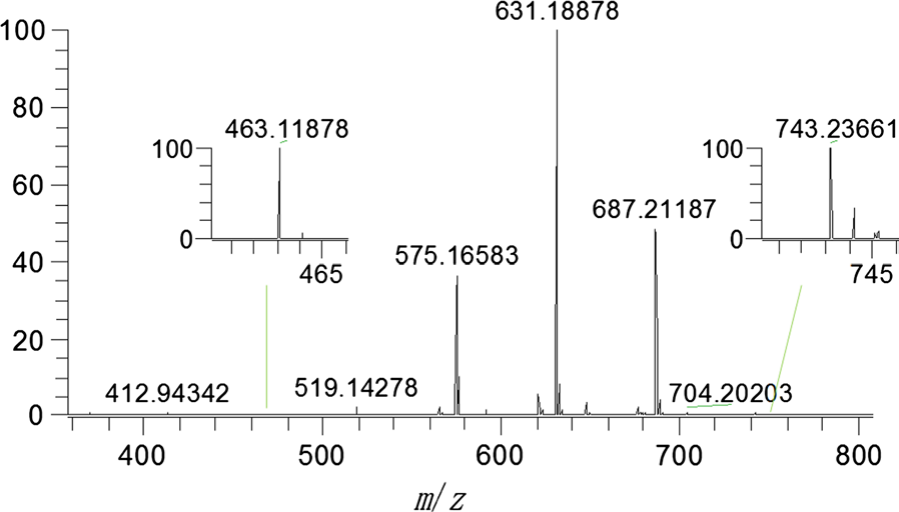
Mass spectra of reaction solution after 1 h under negative ion scanning mode

The synthetic process of CPs was characterized based on electrospray ionization-high resolution mass spectrometry (ESI-HRMS) method [33]. The mass spectra in negative ion scanning mode of the reaction solution after 1 h was shown as Fig. 8, in which m/z = 463.11878, 519.14278, 575.16583, 631.18878, 687.21187, and 743.23611 are the [M + HCOO]^−^ peaks of CP-1, CP-2, CP-3, CP-4, CP-5, and CP-6, respectively. The semiquantative relative contents of the CPs can be calculated based on their integral diagram of mass spectra. In this semiquantative procedure, the yield determination was not based on isolated yields, but it was based on the relative intensiveness of the fragment of CP-6 in the mixtures determined by HRMS.

The visual analysis and variance analysis of the orthogonal test results of propionylation of catalpol were show in Tables 3 and 4, respectively.

**Table 3:** Visual analysis of synthesis process

No.	A	B	C	D	Yield (%)
1	1	1	1	1	4.47
2	1	2	2	2	12.66
3	1	3	3	3	11.45
4	2	1	2	3	26.31
5	2	2	3	1	39.82
6	2	3	1	2	30.06
7	3	1	3	2	68.82
8	3	2	1	3	60.11
9	3	3	2	1	64.91
K1	9.527	33.200	31.547	36.400	
K2	32.063	37.530	34.627	37.180	
K3	64.613	35.473	40.030	32.623	
R	55.086	4.330	8.483	4.557

**Table 4:** Variance analysis of synthesis

Factor	Deviation square sum	Freedom	*F* ^*a*^	*F*_0_^*b*^	Significance
A	4601.945	2	163.497	3.63	Very significant
B	28.147	2	1.000	3.63	–
C	110.649	2	3.931	3.63	Significant
D	35.635	2	1.266	3.63	–
Error	28.15	8	1.002	3.63	–

^a^Variance *F*

^b^Critical value *F*_0_

It can be seen from Table 3 that the factors influence order is A > C>B > D, and the optimal reaction conditions for synthesis of CP-6 are A3B2C3D2, i.e., the molar ratio of acid anhydride to catalpol is 12:1, the reaction temperature is 60 °C, the reaction time is 48 h, and the concentration of pyridine is 20 mg/mL, respectively. Meanwhile, it can be seen from Table 4 that the significant influence factor is A and C, and the Error is not significance which shows that the experiment is reliable.

CP-6 was synthesized under the conditions of A3B2C3D2, with the yield 96.71%.

### Preliminary biological evaluation of designed CP-6

**Fig. 9:**
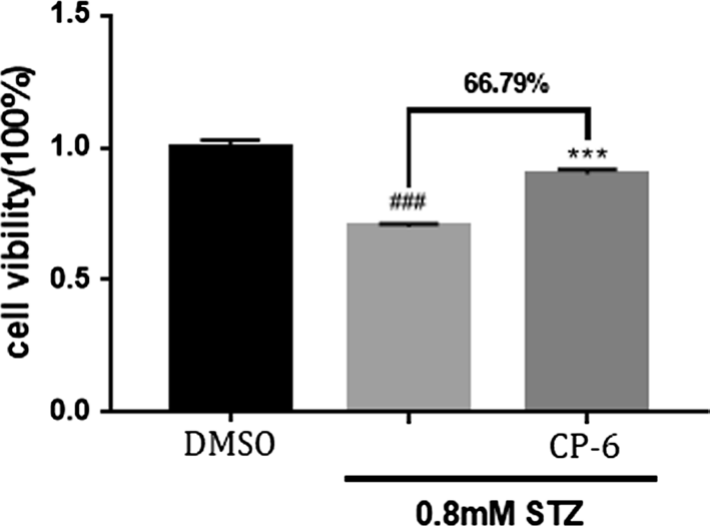
MTT assay for cells viability of CP-6

In the current work, we evaluated the anti-AD effect of CP-6 with activity in preventing against neuronal cell apoptosis by MTT assay. In the assay, STZ was used to induced cell damage in vitro models, which has been widely used to induce glucose metabolism, neuronal apoptosis and tauopathy through oxidative damage. As demonstrated in Fig. 9, 0.8 mM STZ treatment induced an 29.94% ± 1.48% decrease in cell viability for SH-SY5Y, while co-incubation with 20 μM CP-6 effectively reversed STZ-induced reduction of cells viability. These results indicated that CP-6 has protective effects in neurons, and CP-6 has potential antiaging activity.

## Conclusions

The MD results show that the designed CPs maybe are potential antiaging drugs, while the more the number of propionylated hydroxyl groups in catalpol is, the higher of the antiaging activity of the CPs is, and their conformation have effects on their antiaging activity. The designed CPs can be obtained by esterification of catalpol with propionic anhydride in pyridine as solvent and acid banding agent, DMAP as catalyst, and reacted at proper temperature. The optimal reaction conditions for synthesis of CP-6 are molar ratio of acid anhydride to catalpol 12:1, reaction temperature 60 °C, reaction time 48 h, and concentration of pyridine 20 mg/mL, respectively. The MTT assay results indicate that CP-6 has protective effects in neurons and potential antiaging activity.

## Data Availability

The datasets used or analysed during the current study are available from the corresponding author on reasonable request.
